# Development of a guidance to improve allied primary healthcare after acquired brain injury in the Netherlands – a mixed-methods study

**DOI:** 10.1186/s12875-026-03281-x

**Published:** 2026-03-28

**Authors:** Gerbrich Douma, Paulien H. Goossens, Ton Satink, Kitty Jurrius, Maud Graff, Lucas Koester, Hubertus J. M. Vrijhoef

**Affiliations:** 1 IQ Health Science Department, Radboud University Medical Center, Nijmegen, Netherlands; 2 Basalt Rehabilitation Centre, The Hague, Netherlands; 3 Haaglanden Medical Centre, The Hague, Netherlands; 4 Dutch Society of Rehabilitation Medicine, Utrecht, Netherlands; 5 Research Group Neurorehabilitation – Self-regulation and Participation, HAN University of Applied Sciences, Nijmegen, Netherlands; 6 Department of Primary and Community Care, Radboud University Medical Center, Nijmegen, Netherlands; 7 Rehabilitation Department, Radboud University Medical Center, Nijmegen, Netherlands; 8 Dutch Stroke Knowledge Network (KNCN), Utrecht, Netherlands

**Keywords:** Acquired Brain Injury, Stroke, Traumatic Brain Injury, Primary Care, Rehabilitation, Practice Guideline, Occupational Therapy, Physiotherapy, Speech and Language Therapy, Allied Healthcare

## Abstract

**Background:**

The organisation of primary care for individuals with acquired brain injury (ABI) and collaboration in primary care rehabilitation requires improvement. Based on previous research, aimed at improving post-stroke care pathways, this study focuses on the development of guidance for the Netherlands that addresses three key areas: (1) the knowledge and skills of occupational therapists, physiotherapists, and speech and language therapists working in primary care rehabilitation for people with ABI; (2) collaboration in regional primary care networks to deliver appropriate care at the right time and in the right place; and (3) strategies to enhance the visibility and accessibility of suitable therapists for patients with ABI and their professional care providers within primary care.

**Methods:**

A mixed-methods design was employed, guided by the principles of improvement science and in accordance with the Advisory and Expert Group on Quality Standards (AQUA) guideline. The process was structured in three phases: preparation, research, and development. The data were collected through a combination of methods, including interviews and a narrative review. The results of the interviews and narrative review were used to create items for the Delphi study, which were then discussed with expert groups and led to the development of the guidance. Furthermore, input from multiple expert groups was sought throughout the entire process.

**Results:**

Twenty-three interviews were conducted and subsequently analysed. The narrative review was based on 23 relevant articles. The Delphi study reached 95% consensus in two rounds. The need for expertise in ABI treatment, interdisciplinary collaboration, transparent communication, and enhanced accessibility to specialised care, particularly in rural areas, were identified as the most important topics.

**Conclusions:**

A Dutch national guidance aimed at improving primary care rehabilitation for people with ABI was developed successfully through an iterative process. The guidance contains practical tips and advice for allied health professionals.

**Graphical Abstract:**

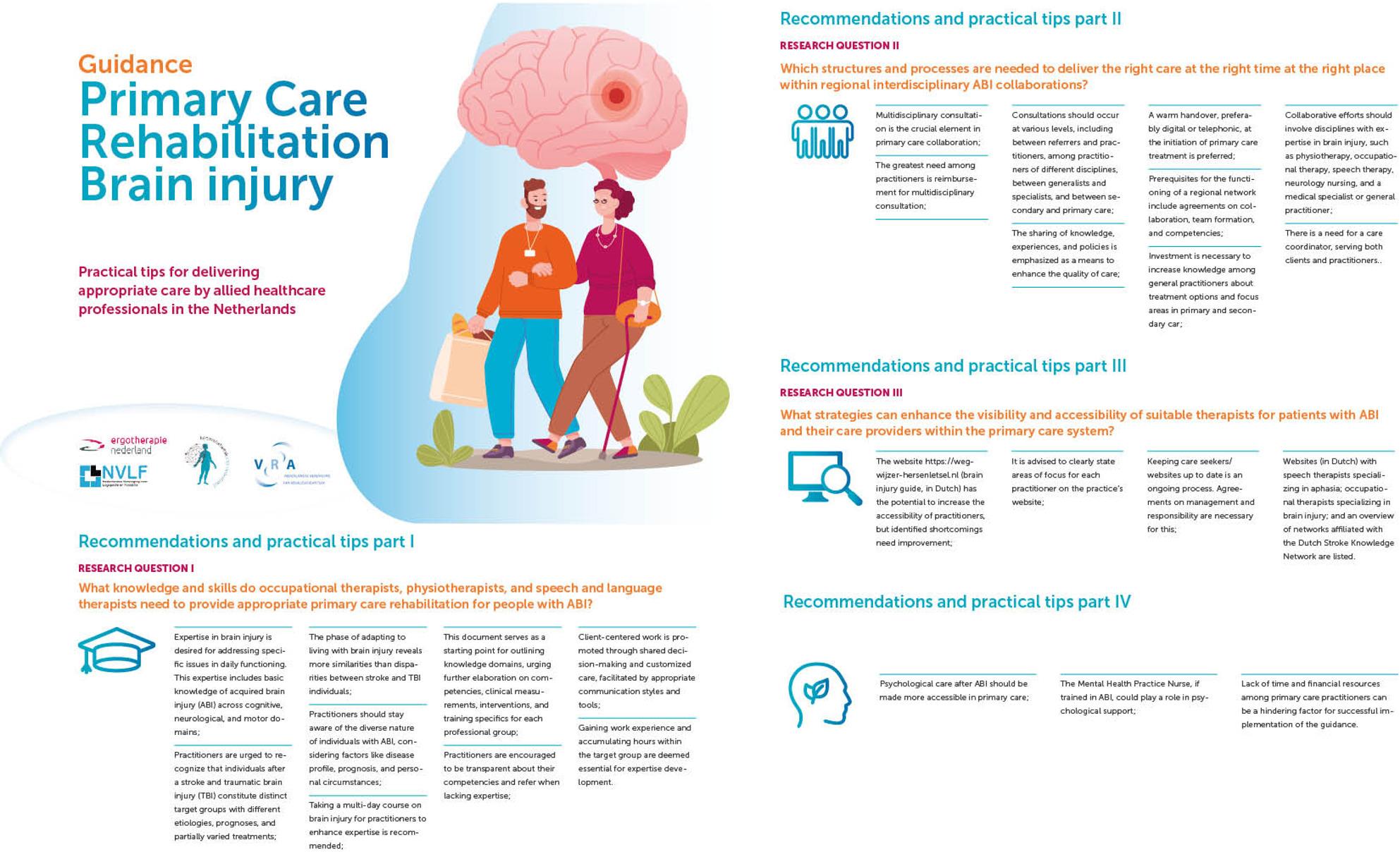

**Supplementary Information:**

The online version contains supplementary material available at 10.1186/s12875-026-03281-x.

## Introduction

The global prevalence of the primary aetiologies of acquired brain injury (ABI), including stroke and traumatic brain injury (TBI), is substantial. In 2021, more than 100 million people worldwide were living with the consequences of stroke [1], and in 2018, the estimated prevalence of traumatic brain injuries was 69 million [2]. The consequences of both diseases can be extensive, resulting in significant disability that necessitates prolonged rehabilitation and support across various healthcare settings [2].

The Stroke Action Plan for Europe [3] places particular emphasis on the organisation of primary care and the strengthening of collaboration in primary care rehabilitation. These elements reflect key aspects of the broader improvement strategy articulated within the document. Recent publications, including the National Clinical Guideline for Stroke [4] and Stroke Rehabilitation in Adults [5], provide recommendations for stroke rehabilitation within community services and primary care. The recommendations primarily focus on the discharge process and community participation. Nevertheless, the translation of these recommendations into clinical practice remains challenging, particularly in countries where primary care rehabilitation is fragmented.

In the Netherlands, rehabilitation following ABI is frequently initiated in hospital or inpatient settings; however, the transition to primary care remains inadequately structured [6]. Regional initiatives that can potentially improve this situation include the ambulant geriatric stroke rehabilitation programme developed by Van Vluggen et al. [7], which integrates inpatient care with home-based aftercare, and the growing attention to the development of post-stroke network care as studied by Van der Veen et al. [8], who emphasised the necessity of regional implementation strategies to facilitate the integration of rehabilitation into primary care following stroke. Arwert et al. [6] conducted a comprehensive mapping of formalised Dutch primary care stroke networks, providing detailed insights into their organisational structures and operational processes. The findings of the study revealed the existence of multiple regional networks designed to deliver appropriate care in the right setting post-stroke. However, a paucity of consensus exists regarding the structure, processes, and roles within these networks, and national coverage remains incomplete. Furthermore, patients and referrers frequently encounter difficulties in locating suitable therapists, partly because the absence of clear guidelines on the required competencies makes it challenging to determine which primary care providers are adequately qualified. Despite the existence of monodisciplinary guidelines that delineate relevant knowledge and skills, these competencies are not consistently translated into primary care practice, as these guidelines are not uniformly implemented or used to identify which primary care providers possess the required expertise. To address these gaps, Arwert et al. [6] recommended the establishment of multidisciplinary stroke networks comprising allied health professionals, specifically occupational therapists, physiotherapists and speech and language therapists. Additionally, it is imperative that these networks prioritise interprofessional collaboration, continuous quality improvement, and enhanced visibility among users.

These gaps in knowledge and primary care practice underscore the imperative for national, evidence- and consensus-based guidance to support allied health professionals in delivering coordinated, person-centred rehabilitation within the community. It is imperative that interdisciplinary collaboration is strengthened and that the visibility and accessibility of services in primary care are improved to ensure that people with ABI receive the right care at the right time and in the right place.

The present study was initiated to address these identified gaps in the literature by developing a national guidance document. The overarching aim of this document is to support allied health professionals in the delivery of coordinated, person-centred rehabilitation in primary care settings. The overarching objective of the guidance is to enhance interdisciplinary collaboration, optimise service accessibility, and ensure that individuals receive the right care at the right time and the right place.

The research questions (RQs) were formulated based on a preliminary literature review, expert consultations, and stakeholder input from professional associations and patient organisations. They reflect the most pressing challenges identified in current practice and are designed to inform the structure and content of the national guidance:

1. What knowledge and skills do occupational therapists, physiotherapists, and speech and language therapists need to provide appropriate primary care rehabilitation for people with ABI?
2. Which structures and processes are needed to deliver the right care at the right time at the right place within regional interdisciplinary ABI collaborations?
3. What strategies can enhance the visibility and accessibility of suitable therapists for patients with ABI and their healthcare providers within the primary care system?

In this paper, we report on the process of developing the guidance, the research findings, and the content of the national guidance.

## Methods

### Study design

The study methods were based on the principles of improvement science, as described by Berwick [9], aiming at quality improvement in healthcare and meaningful stakeholder involvement, using an iterative, stakeholder-driven development process across all study phases.

Mixed-methods research was applied for the data collection, following the principles of the Advisory and Expert Group on Quality Standards (AQUA) guideline of the Dutch National Healthcare Institute [10]. This guideline provides methodological guidance for the development of healthcare quality standards, care modules, organisational descriptions, and clinical guidelines in the Netherlands. It serves as a structured framework that relates to parts of the care process and defines what is necessary to deliver good care from the client’s perspective [10]. The AQUA Guideline was chosen because it is the nationally endorsed framework for developing quality standards in Dutch healthcare, ensuring alignment with existing policy structures and professional practices. For international readers, AQUA aligns with internationally recognised approaches for guideline development, such as the NICE guideline development principles [11] or the AGREE II framework [12], while placing a specific emphasis on client-centred care and co-design. Applying its principles enabled an experience-based co-design approach, contributing to a more holistic understanding of the research objectives.

**Fig. 1:**
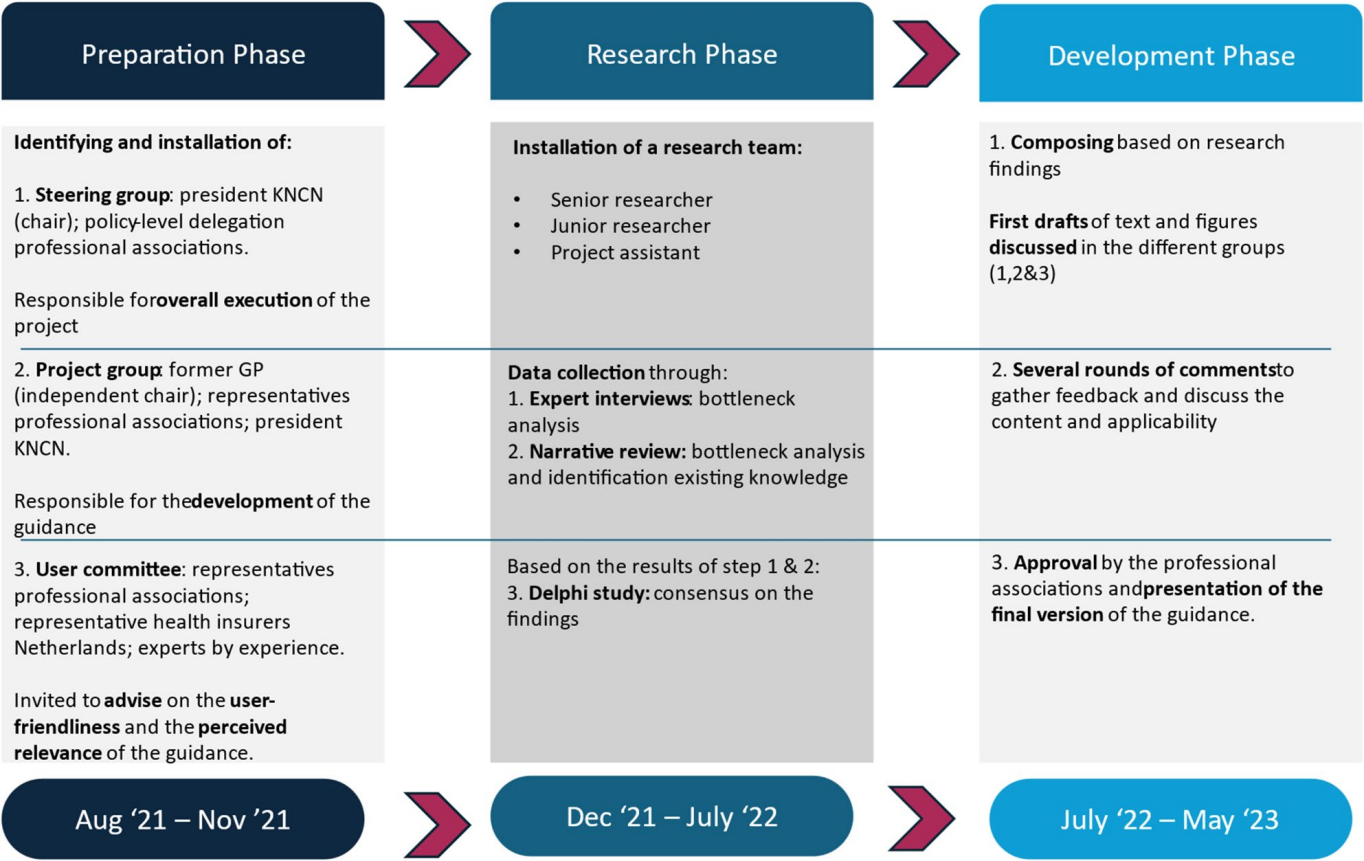
A description of the three phases employed to create the guidance

Expert groups were utilised during all phases of this project; they comprised representatives from the Dutch professional associations of occupational therapists, physiotherapists, speech and language therapists, and rehabilitation physicians, in addition to ABI survivors who served as experts by experience. The aforementioned expert groups were divided into a steering group, a project group, and a user committee. The project was led and organised by the Dutch Stroke Knowledge Network (in Dutch: *Kennisnetwerk CVA Nederland* [KNCN]). A grant for the development of the national guidance was received from the Brain Foundation Netherlands (grant number: DR-2020-00379). The development of the guidance comprised three phases, as presented in Fig. 1.

### Data collection and analysis

Data were collected and analysed [1] to explore current provision and to identify bottlenecks in primary care rehabilitation by allied health professionals and [2] to study the content and topics to be addressed in the guidance.

### Interviews

A maximum of 25 semi-structured expert interviews, or a smaller number until data saturation was reached, were planned to identify bottlenecks in the current provision of primary care rehabilitation for people with ABI. The aim was to include people with ABI (target group 1), therapists (occupational therapists, physiotherapists, and speech and language therapists) and rehabilitation physicians (target group 2), and a wide range of other healthcare providers is also involved, including general practitioners (GPs), psychologists, representatives of healthcare networks, and specialists in the field of outpatient rehabilitation (target group 3). The inclusion of target group 3 was driven by the objective of acquiring a comprehensive array of information. This sampling strategy ensured that perspectives from both people with ABI and a range of professional stakeholders could be explored. The interviewees were recruited through the KNCN and the networks of the stakeholders in the expert groups employed in this study. Participants were provided with an information letter written at a B1 level. In the event of any subsequent queries, the researcher’s contact details were made available.

For people with ABI, they or their spouse had to be able to communicate verbally. For allied health professionals and rehabilitation physicians, the aim was to include two participants from each discipline. The group of other healthcare providers was selected based on availability and a wide range of backgrounds. The topic guide (see Supplementary Material 1 Appendix I) for the interviews was developed in collaboration with the participants of the steering and project groups. The questions focused on personal experiences with therapists, the desired skills and knowledge of therapists, collaboration in primary care rehabilitation, the visibility and accessibility of therapists, and barriers and facilitators in developing and implementing the guidance. The interviews were conducted by the junior researcher (GD) via Microsoft Teams. Verbal informed consent was obtained at the start of each interview.

A hybrid thematic analysis [13, 14] was employed, combining a deductive structure—based on three guiding research questions—with inductive coding of participants’ statements. This approach enabled a focused yet data-driven identification of bottlenecks within the study’s scope. All interviews were transcribed verbatim by the junior researcher (GD) and read thoroughly for familiarisation (step 1). The interviews were coded using open coding (step 2). Subsequently, the extent to which the interviewees’ statements were relevant to and addressed the RQs was examined to generate themes (step 3). Then, the themes were reviewed by further identifying which overarching themes and subthemes could be derived from the coded interview data, and which new themes were introduced by the interviewees (step 4). The senior researcher (HV) reviewed the initial findings of the thematic analysis. The identified themes were discussed between the two researchers and finally defined (step 5). The topics and themes that emerged from the interviews were used to build up the list of items for the Delphi study (step 6).

### Narrative review

To synthesise national and international literature on allied health professionals working in primary care with individuals with ABI, a narrative review was conducted. Insights from the review were used to inform the development of a Delphi study. A narrative approach was chosen to allow for flexible synthesis across diverse study types and formats. To ensure transparency and methodological rigour, the review was structured in accordance with the SANRA (Scale for the Assessment of Narrative Review Articles) criteria [15], which emphasise clarity of objectives, relevance of the topic, adequacy of the literature search, appropriate referencing, and coherence of synthesis.

The objective of the review was to explore three interconnected themes: (a) the skills and knowledge required by allied health professionals when working with people with ABI; (b) the organisation and delivery of care, including collaboration within network-based care; (c) the accessibility and visibility of appropriate therapists from the perspectives of both clients and referrers.

A structured literature search was conducted in national and international knowledge repositories, including PubMed, CINAHL, PEDro, the Kennisnetwerk CVA Nederland (Dutch Stroke Knowledge Network) repository, and the databases of relevant Dutch professional associations (e.g., the Dutch Society of Rehabilitation Medicine [VRA], the Royal Dutch Society for Physical Therapy [KNGF], Ergotherapie Nederland [EN], and the Dutch Association for Speech and Language Therapy and Phoniatrics [NVLF]) between December 2021 and March 2022. The search strategy was developed collaboratively by two researchers (GD and HV), using predefined inclusion criteria: full-text availability, publication in Dutch or English, and relevance to the three guiding research questions. Although no strict publication date limit was set, the search focused on contemporary literature relevant to current primary care practice. Additional sources were identified through expert interviews, reference list screening, and analysis of documents related to the grant application. All search results were documented in a logbook and organised by author and publication date.

Titles and abstracts were independently screened by both researchers (GD and HV). Full-text review was conducted to assess relevance to the research questions. Studies were excluded if they lacked pertinent information on allied healthcare in primary care or did not focus on ABI or stroke populations. No formal assessment of the level of evidence was conducted, as the review aimed to provide a broad thematic overview rather than a weighted synthesis of study quality. The selected articles were summarised in a table including author, year, title, and relevance to the research questions.

### Delphi study

The items were selected by the researchers (GD and HV) and the project assistant (LK) for review within the Delphi study [16, 17]. The items were derived from three sources: the themes derived from the interviews, the topics identified during the narrative review, and a discussion and proposal from the project group to the steering committee. Each source was treated with equal importance, and no differential weighting was assigned during item selection.

Three rounds were planned: two online, using SurveyMonkey, followed by a live meeting to facilitate discussions. If sufficient consensus was reached prior to the conclusion of the designated number of rounds, a decision could be made to eliminate one or more of the remaining rounds. The Delphi panel was formed by a maximum of 35 participants who applied as experts in the field of ABI, as suggested by the project and steering groups. In line with Berwick’s improvement science principles [9], the Delphi method was used to generate practice-oriented knowledge through iterative consensus-building. Details on participant characteristics are provided in the Results section.

In the Delphi study, items were rated in three categories based on the RQs. The topics were rated on a 9-point Likert scale, with a score of 7, 8, or 9 being considered (highly) relevant and therefore included in the analysis as a positive rating, and 1, 2, or 3 being considered (totally) irrelevant and included in the analysis as a negative rating. Scores of 4, 5, and 6 were interpreted as neutral or ambiguous, indicating uncertainty or moderate relevance, and were not included in either the positive or negative category. Additionally, the participants were requested to compile the top three items they deemed most important. The top three was subjected to analysis and used to established the hierarchy of topics within the guidance. As recommended in a previous study [16], the cut-off value for reaching a consensus was 75%. Besides the cut-off value, another criterion for reaching consensus was that a score of 1–3 was given by less than 15% of the participants [18].

### Composing the guidance – expert opinion

Throughout the project, multiple coordination and discussion meetings were held with the steering group, the project group, and the user committee. A detailed overview of the number of meetings per group is presented in Table 6. After the research phase, a draft version of the guidance was written by the junior researcher (GD), with input from the various groups. In an iterative process with input from the steering group, the project group, and the user committee, the draft was modified by the researchers (GD and HV) and the project assistant (LK) and submitted to the boards of the professional associations for approval.

### Ethical considerations

The study followed all applicable ethical and legal standards in the Netherlands. These comprised the Medical Research Involving Human Subjects Act (WMO), the Medical Treatment Contracts Act (WGBO), the Declaration of Helsinki, and the Netherlands Code of Conduct for Research Integrity [19, 20]. As the study did not involve medical interventions or the collection of data that could be considered invasive or burdensome, it did not fall under the scope of the WMO, and therefore formal ethical approval was not required.

Compliance with the WGBO was ensured by informing all participants about the purpose of the study, the voluntary nature of participation, confidentiality safeguards, and their right to withdraw at any time. Data were processed and stored in accordance with national data protection standards and institutional procedures.

## Results

### Interviews

In total, 23 semi-structured interviews were conducted between January and March 2022. The interviewees included eight individuals with ABI, including two interviews in which the person with ABI and their spouse participated. Additionally, eight allied health professionals participated, including occupational therapists (*n* = 2), physiotherapists (*n* = 2), speech and language therapists (*n* = 2), and rehabilitation physicians (*n* = 2). Furthermore, seven healthcare professionals with various (medical) backgrounds were interviewed. These included a general practitioner (GP) (*n* = 1), psychologist (*n* = 2), a neurology nurse practitioner (*n* = 1), and representatives of an ambulatory brain treatment programme (*n* = 1), a Parkinson network (*n* = 1), and a regional neurology network (*n* = 1). The baseline characteristics of the interviewees are represented in Tables 1 and 2. On average, the interviews lasted 42 min (minimum = 35 and maximum = 57). This sample composition allowed perspectives from people with ABI and professional stakeholders to be considered alongside one another.

**Table 1:** Baseline characteristics of the interviewees: expert by experience

Target group/interviewee	Age range (years)	Nature of the condition	Time since condition (years)
*Target group 1*			
Expert by experience I	60–65	Traumatic brain injury	2
Expert by experience/spouse II	65-70 50-55	Stroke	7
Expert by experience III	60–65	Stroke	12
Expert by experience IV	45–50	Stroke due to a brain tumour	14
Expert by experience V	50–55	Stroke	8
Expert by experience VI	25–30	Stroke	3
Expert by experience/spouse VII	55-60 55-60	Stroke	4
Expert by experience VIII	65–70	Stroke	3

**Table 2:** Baseline characteristics of the interviewees: professionals

Target group/interviewee	Experience in practice (years)
*Target group 2*	
Occupational Therapist	19
Occupational therapist	13
Physiotherapist	14
Physiotherapist	28
Rehabilitation physician	29
Rehabilitation physician	22
Speech and language therapist	11
Speech and language therapist	8
*Target group 3*	
Clinical psychologist	2
Clinical psychologist and lead practitioner ambulatory brain treatment programme	4
General practitioner for cardiovascular diseases	8
Neurology Nurse Practitioner	17
Representative of an ambulatory brain treatment programme	6
Representative of a Parkinson network	6
Representative of a regional neurology network	5

**Fig. 2:**
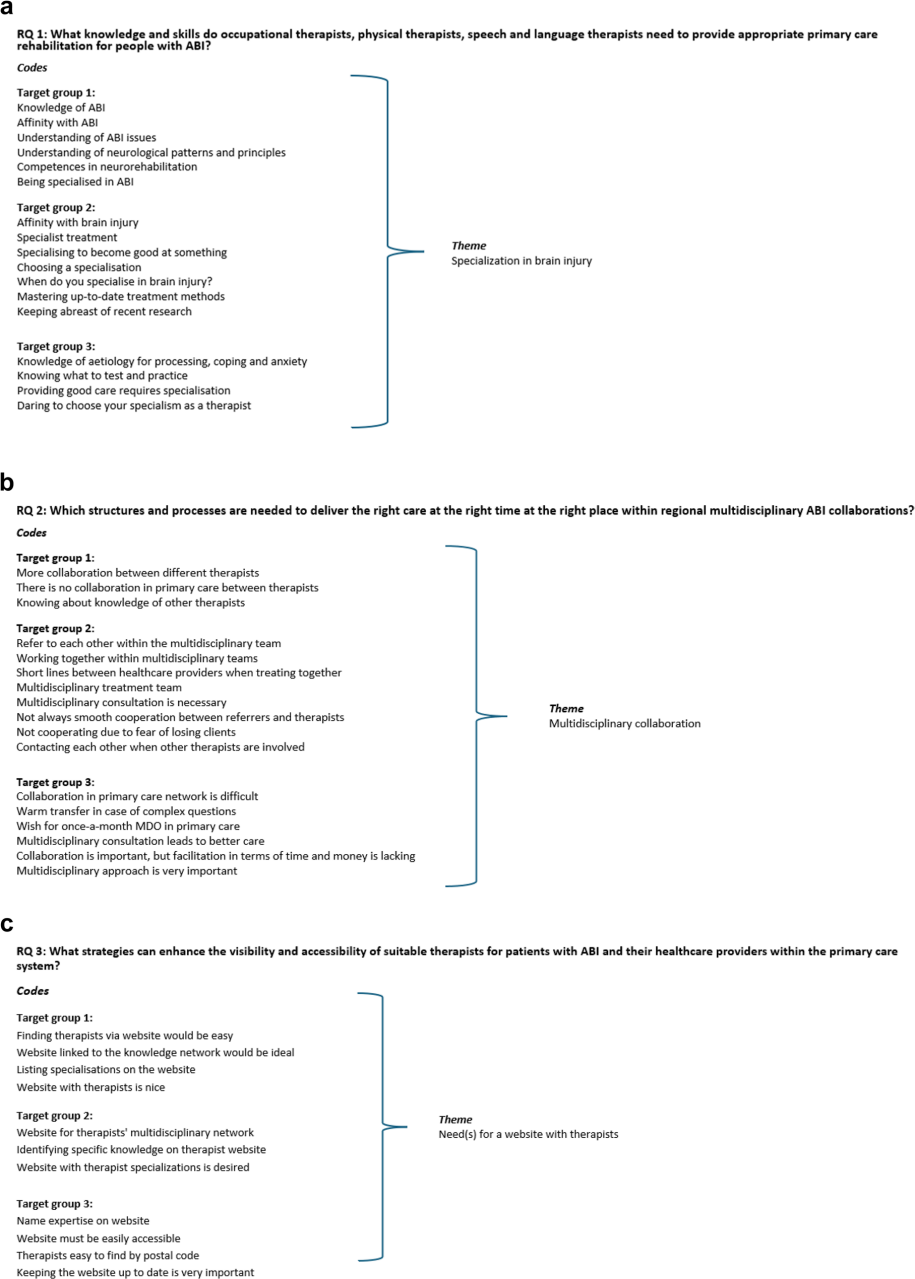
Illustrative examples of how inductively coded statements were grouped into themes in response to each research question (RQ). **a** Codes and theme related to RQ1; **b** Codes and theme related to RQ2; **c** Codes and a theme related to RQ3

The thematic analysis of the interviews yielded 605 codes, which were divided into 66 themes focused on the RQs. For RQ 1, there were 180 codes across 27 themes; for RQ 2, there were 158 codes and 21 themes; and for RQ 3, there were 171 codes and seven themes. Additionally, 96 codes were divided into 11 themes that were not directly related to the RQs. The themes were translated into items, which were used for the Delphi study. Figure 2 illustrates the progression from inductive coding to theme development, providing examples for each research question.

### Narrative review

The preliminary search yielded a total of 804 articles from national and international databases. After a thorough screening of titles and abstracts, 24 publications were considered potentially relevant. Following full-text screening, during which the predefined exclusion criteria—such as lack of focus on allied healthcare in primary care or absence of ABI/stroke populations—were applied, 14 articles were deemed eligible for inclusion. An additional 9 sources were identified through reference lists, expert input, and national guidelines not indexed in the searched databases. This resulted in a total of 23 articles included in the review, comprising 10 Dutch sources (Table 3, references 6, 7, 21–28) and 13 international sources (Table 4, references 3, 29–40). Dutch and international studies were presented separately because the Dutch papers provided essential context‑specific insights into the organisation of primary care and rehabilitation pathways in the Netherlands, whereas the international literature contributed broader evidence and best practices that informed the overarching themes.

**Table 3:** Details for the Dutch studies included in the narrative review

Author and reference number	Year of publication	Key findings in relation to research question 1: ‘Therapists’ knowledge and skills’	Key findings in relation to research question 2: ‘Right care at the right time within regional collaboration’	Key findings in relation to research question 3: ‘Visibility and accessibility of therapists’
Van Heugten et al. [19]	2021	Organise specific ABI training (e.g., focus on the difference between ABI after stroke or TBI).	Form brain injury networks. Strengthen regional networks by getting to know each other and each other’s expertise, and involve coordinators/ consultants, post-care options, and experiential experts.	Provide recommendations for increasing visibility.
Vluggen et al. [7]	2020		There is no established homecare rehabilitation in the Netherlands.	
Stiekema et al. [20]	2020	Professionals need to work in a client-centred and demand-driven manner.	It is challenging to receive the right support at the right time. There is a need for a case manager for brain injury.	
Van der Veen [8]	2019	Client-centred treatment is crucial. Providing treatment at home has advantages in terms of customisation and generalisation. Professionals need education in neurology and should regularly treat post-stroke clients. There should be transparency in expertise.	Professionalisation should be promoted through coordinated aftercare by a ‘key agent’ and structured communication options. Interprofessional collaboration is lacking, and there are insufficient opportunities for consultation. The recommendation is to develop region-specific strategies for successful implementation.	The type of therapy that can be provided depends on the client’s insurance. It is unclear which professional possesses specific knowledge, and there is a lack of information on where to find professionals – an overview is missing.
Arwert et al. [21]	2019		A lack of time and financial resources is the biggest obstacle to primary network care. Short lines of communication and a ‘bottom-up’ structure are key success factors.	
Borcherts [22] * This report serves as the catalyst for the present research.	2018	It is unclear what knowledge and skills therapists working in the primary care setting need to provide appropriate care.	Finding the right care at the right time is a challenge.	There is a desire to increase the visibility and accessibility of therapists for referrers and patients.
Minkman [23]	2013	Generalists should be available where possible, and specialists should be available where necessary.	Within chain care, attention is given to the individual needs of the client. Case management should be provided for complex questions.	
Veerbeek [24]	2017	An additional point from the guideline is to treating stroke clients regularly. There should be screening for the risk of falls.		
Berns [25]	2015	An additional point from the guideline is that adaptation is key for written text for individuals with aphasia.		
Steultjens [26]	2013	Additional points from the guideline are: map the coping style and pay attention to the burden on the caregiver.		

**Table 4:** Details for the international studies included in the narrative review

Author and reference number	Year of publication	Key findings in relation to research question 1: ‘Therapists’ knowledge and skills’	Key findings in relation to research question 2: ‘Right care at the right time within regional collaboration’	Key findings in relation to research question 3: ‘Visibility and accessibility of therapists’
Pedersen [27]	2021		Discharge summaries could have the potential to serve as a tool for collaboration and knowledge transfer.	
Di Carlo [28]	2021	There are knowledge gaps.	Important issues include transitions in healthcare, access to resources and a healthcare team, lack of support, and health insurance.	
Walder [29]	2020	There is a need to be understood by the therapist, to respond to the patient’s needs, to involve them in decision-making, and to provide information.		
Juckett [30]	2020			Although there are evidence-based practices for occupational therapists regarding stroke rehabilitation, attention should be given to implementation strategies to apply them effectively.
Parappilly [31]	2019		In the home setting, there is room for providing information and/or secondary prevention. There is no room for this during the acute phase.	
Norrving [3]	2018	One of the formulated objectives is: what are the minimum training criteria for stroke experts (physicians, nurses, or therapists)?		
Lou [32]	2016		*Home is a favourable place for early rehabilitation when the right support can be provided.*	
Martinsen [33]	2015	Personalised care is missed.	Access to follow-up is challenging.	
Dworzynski [34]	2015		There are significant differences in national rehabilitation programmes; uniformity is necessary. A multidisciplinary rehabilitation team consists of physicians, nurses, physiotherapists, occupational therapists, speech therapists, clinical psychologists, rehabilitation assistants, and social workers. The transition between the hospital and home must be comprehensive.	
Tholin [35]	2014	Evidence-based practice is important in stroke rehabilitation. The offerings should be tailored to the client.	Communication about follow-up is crucial.	
Carlsson [36]	2010		To provide safe care, there must be standardised information recording and transmission.	
Venkatasubramanian [37]	2008		The family physician is seen as the process guardian after discharge when a specialist is no longer involved. A stroke nurse can take on the role of a care coordinator.	
Ski [38]	2007	Information from the hospital is desirable to better prepare the caregiver for what lies ahead.	The importance of follow-up is emphasised.	

The selected articles provided relevant insights in relation to the three guiding research questions. Relevant information related to the research questions was extracted and narratively synthesised, focusing on descriptive integration rather than formal thematic coding. Extracted information was used to inform the development of items for the Delphi study. In addition, key findings from the literature served to substantiate the recommendations formulated in the guidance document.

### Delphi study

Of the 35 people invited to participate in the online survey, 26 responded and 25 completed the survey. They were expert by experience (*n* = 6), occupational therapists (*n* = 2), physiotherapists (*n* = 6), speech and language therapists (*n* = 7), rehabilitation doctors (*n* = 3), and a representative from an ambulatory brain treatment programme (*n* = 1).

At the outset of the Delphi study, 74 distinct items (see Supplementary Material 1 Appendix II) were subjected to evaluation. The 74 items under consideration were derived from a comprehensive discussion within the various expert opinion groups. These items were selected based on a longlist of items derived from the analysis of interview data and a review of the extant literature. In the initial phase of the Delphi study, which took place between 2 and 13 May 2022, 71% of the participants completed the survey. The mean time taken to complete the survey was 20.4 min (standard deviation 7.2). Following this initial phase, a consensus was reached on 95% of the items. In addition to evaluating the items, the participants were encouraged to contribute any missing topics for consideration in the survey. Four suggestions were received, all of which were directly incorporated into the second phase of the Delphi study.

In June 2022, the 25 respondents from the initial round were invited to participate in the second phase of the study. Of the participants, 17 (68%) completed the questionnaire in its entirety, with an average completion time of 7.2 min (standard deviation 3.6). In this second phase of the study, only the eight items for which no consensus had been reached in the initial round were subjected to evaluation, and a consensus was reached for 75% of these items. A total of 78 unique items were considered, and a sufficient consensus was reached for 97% of these items within two rounds. As a result, the scheduled live meeting was cancelled.

Table 5 presents the primary findings as identified by the Delphi panel in their top three selections.

**Table 5:** Top 3 items based on the first round of the Delphi study, for each problem area and target group

Main items of the Delphi study	Number of times mentioned in the top 3
*Therapists’ knowledge and skills*	
Experience experts (*n* = 6)	
1. Specific training in brain injury	4
2. Providing customised care	3
3. Multi-/interdisciplinary collaboration	2
3. Coping styles	2
Practitioners (*n* = 19)	
1. Working based on the client’s needs	8
2. Specific training in brain injury	7
2. Providing customised care	7
2. Multi-/interdisciplinary collaboration	7
2. Specialisation in brain injury	7
*Right care at the right time within regional collaboration*	
Experience experts (*n* = 6)	
1. Sharing knowledge	3
2. Experiential networks focused on exchanging experiences	2
2. Formal networks at the policy level	2
2. Live transfer between therapists (warm handover)	2
2. Making agreements about collaboration	2
Practitioners (*n* = 19)	
1. Financial coverage of multi-/interdisciplinary meetings	9
2. Multidisciplinary collaboration	8
2. Interdisciplinary collaboration	8
3. Sharing knowledge	5
3. Acting as a ‘hub’, coordinator of care	5
*Visibility and accessibility of therapists*	
Experience experts (*n* = 6)	
1. Referral from hospitals/rehabilitation centres to primary care	6
2. Brain Injury Guide (website with therapist listings)	5
3. Need for a knowledge base (website with a collection of knowledge)	3
Practitioners (*n* = 19)	
1. Referral from hospitals/rehabilitation centres to the primary care	17
2. Brain Injury Guide (website with therapist listings)	9
3. Need for a knowledge base (website with a collection of knowledge)	8

### Composing and finalising the guidance – expert opinion

The number of coordination and discussion meetings of the different groups during the preparation, research, and development phases is illustrated in Table 6.

**Table 6:** Number of meetings for each expert opinion group

Expert group	Number of meetings
Steering group	12
Project group	7
User committee	6

The study design included representation from the various allied health professional associations in all working groups and phases of the study. All professional groups were represented in the project group, steering group, and user committee through delegates appointed by their respective professional associations, and professionals from each discipline also participated in the interviews and Delphi rounds on a personal basis. During the research, it became evident that there was a desire among the professionals to confirm their practices in accordance with the requisite quality standards. The interviewed therapists sought to establish defined criteria, such as competencies and caseloads, which would result in the inclusion or exclusion of therapists. The professional associations, however, emphasised that their mandate is to represent the full diversity of their members, which limits their ability to endorse criteria that formally distinguish subgroups of therapists.

During the development phase, all professional associations were invited to solicit feedback from their respective constituencies. In the finalisation phase of the guidance (January 2023), the association of physiotherapists decided to withdraw from participation, independently of the researchers. The association stated that recommendations concerning physiotherapy expertise should originate from within the profession itself, and that endorsing external recommendations would not align with its representational role. This decision did not affect the participation of physiotherapists throughout the project nor the inclusion of physiotherapy perspectives in the guidance, and individual physiotherapists remain free to use the guidance in practice.

Following this withdrawal, the associations of occupational therapists, speech and language therapists, and rehabilitation physicians chose to proceed with and actively support the development of the guidance. The steering group decided to continue the process, and the modified version of the guidance was submitted to the boards of the professional associations of occupational therapists, speech and language therapists and rehabilitation doctors who accredited the final version of the guidance. In May 2023, the national guidance was presented to the public.

### Content of the guidance

Data obtained from the interviews and literature review informed the Delphi study. The Delphi results together with insights from the expert opinion discussions, were translated into the various chapters of the guidance. The overall structure of the guidance reflects the three guiding research questions. 

#### Skills and knowledge required of allied health professionals

The initial part of the guidance describes the skills and knowledge required of therapists treating individuals with ABI. The research phase did not yield definitive recommendations regarding specific educational courses or training programmes, and expert groups confirmed that accrediting educational activities fell outside the scope of this project. Therefore, knowledge requirements are described in general terms. The guidance emphasises the importance of developing expertise through practical experience and education, transparency regarding one’s professional capabilities, the use of shared decision‑making, and communication tailored to the client’s situation. A preliminary framework of knowledge domains is included, with the potential for further development by professional associations.

#### Interdisciplinary collaboration in primary care

Interdisciplinary collaboration is identified as a key component of high‑quality care.

To support and strengthen regional collaboration in primary care, the guidance outlines the importance of interdisciplinary cooperation at different organisational levels. Reimbursement for interdisciplinary consultations is described as essential for the delivery of high‑quality care. Formal agreements regarding collaboration and transitions between care providers are presented as important mechanisms for ensuring continuity. The guidance further identifies the need for a care coordinator with knowledge of the regional social support landscape to facilitate coordination.

#### Visibility and accessibility of allied health professionals

The guidance highlights strategies to improve the visibility and accessibility of therapists. Informal networks play an important role in referrals to allied health professionals. Accessibility for healthcare providers (e.g., neurologists and general practitioners) and for patients can be improved by using a database with up‑to‑date information about therapists. Current regional networks cover only part of the country, and even within these networks visibility remains limited. A shortage of specialised healthcare providers, especially in rural areas, further contributes to challenges in accessibility.

### Additional topics emerging from the research

The research also identified several topics beyond the three main research questions. These include the need for knowledge about the differences between stroke and TBI, the demand for psychological care in primary care settings for people with ABI, and the requirement for additional time and resources to implement primary care rehabilitation for individuals after stroke or TBI.

An item‑by‑item summary of the guidance is provided in Supplementary Material 1 Appendix III.

## Discussion

This article presents interdisciplinary solutions to address gaps in primary care rehabilitation for individuals with ABI, aiming to support allied health professionals in delivering integrated and person-centred care. The relevance of these solutions lies in the rising prevalence of ABI, the relatively high costs and limited capacity within secondary care, and the preference of individuals with ABI to receive rehabilitation as close to home as possible. The findings reflect the predefined research questions guiding this study, each addressing a critical aspect of integrated rehabilitation in primary care: professional competencies, interdisciplinary collaboration, and service accessibility.

One key finding of this study is that ABI-related expertise and an affinity with the target population are essential for the provision of effective primary care rehabilitation. This need was expressed not only by allied health professionals but also by people with ABI, who emphasised the importance of working with therapists who understand the long‑term consequences of an ABI and can tailor their approach to the individual. By incorporating perspectives from both people with ABI and professional stakeholders, the study reduces the risk that this finding merely reflects the views of the professionals involved. Rather, it highlights a shared view across stakeholder groups that specialised knowledge and affinity are important for supporting individuals with ABI throughout the recovery trajectory.

This is consistent with Elbaum [39], who describes how interdisciplinary expertise remains critical beyond the acute phase, particularly when psychosocial support and reintegration become central. Smith et al. [40] similarly emphasise the value of trained professionals who combine technical knowledge with personal engagement, noting that stroke survivors felt safer and more motivated when supported by instructors who “knew what they were talking about.” These insights reinforce the importance of sustained, specialised care within primary care settings, and underline the relevance of equipping primary care professionals with targeted ABI knowledge and sensitivity.

Another important insight concerns cooperation and communication between diverse professionals and within networks, which are crucial for addressing the needs of individuals with ABI and fostering interprofessional collaboration. In addition to highlighting the need for professional expertise, Elbaum [41] emphasises that interdisciplinary teamwork and effective communication are essential throughout the rehabilitation process, enabling professionals to respond to the complex and evolving needs of ABI patients. Paxino et al. [39] further demonstrate that successful communication within rehabilitation teams depends on role clarity, mutual trust, and shared goals, and must be actively cultivated to support coordinated care. Taken together, these insights underline the importance of strengthening specific collaborative competencies, such as clear role negotiation, shared decision‑making, effective communication, and the ability to work within regional networks, to support coordinated and network‑based primary care rehabilitation.

The potential of digital infrastructure to improve accessibility also emerged from the data. While Pearce et al. [42] primarily focus on digital interventions within rehabilitation, their findings suggest that well-designed digital platforms can enhance accessibility and user engagement. In line with this, regularly updated regional websites may serve as practical tools to improve the visibility of skilled therapists and support informed referral processes, thereby strengthening the connection between patients and appropriate care providers.

Among the additional topics that emerged beyond the three research questions, two themes stood out because they were consistently raised across stakeholder groups and have direct implications for primary care practice. These include the importance of distinguishing between different forms of ABI in primary care and the incorporation of psychological counselling as part of rehabilitation. Research shows that psychosocial issues, such as anxiety and depression, often persist beyond the acute phase [43], and that targeted psychological support can improve participation and quality of life [44].

The guidance developed in this study provides general recommendations for primary care rehabilitation and serves as a foundation to reduce unwanted practice variation in the treatment of individuals with ABI in the Netherlands. Our findings align with the National Institute for Health and Care Excellence (NICE) guideline ‘Stroke rehabilitation in adults’ [5], particularly regarding the composition of interdisciplinary teams, person-centred practice, communication, and (re)referral. For example, the emphasis on ABI-related expertise and affinity with the target group aligns with the NICE Stroke Rehabilitation in Adults guideline (NG236), which states that professionals should take the guideline fully into account alongside the individual needs, preferences, and values of their patients. Similarly, the importance of structured collaboration and clear referral pathways resonates with NICE’s recommendations on interdisciplinary coordination. As such, this study incorporates relevant aspects of the NICE guideline within the Dutch primary care context and offers concrete steps for quality improvement.

### Strengths and weaknesses

A methodological point of discussion during both the research and development phases of the guidance is the inclusion of participants with various forms of ABI. According to a literature review [45], the recommendations for rehabilitation of people with different forms of ABI contain more similarities than differences. Nonetheless, people with various forms of ABI develop differently over time [46, 47], reflecting the diversity of perceived limitations. The inclusion of people with mild TBI (after concussion or contusion of the brain) is another point of discussion. Interviews and expert opinions revealed that rehabilitation of people with mild TBI in primary care tends to focus more on experienced cognitive limitations and often follows different care pathways. As a result, the group discussions led to a deliberate decision to focus the guidance on individuals with stroke and those with moderate to severe TBI with objectively confirmed brain injury on neuroimaging, rather than mild TBI where no structural damage is usually visible on CT or MRI.

While this scope limits the generalisability of the recommendations to the broader ABI population, it enhances their specificity and practical relevance. Importantly, the guidance combines stroke and moderate to severe TBI with imaging-confirmed structural brain injury as a unified target group, based on shared rehabilitation needs and the fact that these individuals are typically treated by the same professionals in routine primary care practice. This alignment strengthens the applicability of the recommendations and supports integrated care delivery within existing clinical structures.

A strength of this study is its mixed-methods design, following the AQUA principles [11]. Throughout the research and development process, multiple stakeholder groups, including people with ABI, informal carers, allied health professionals, and rehabilitation physicians, were involved in iterative discussions on the interview findings, the Delphi items, and the draft guidance content. This approach introduced co‑creative elements, as stakeholders contributed to shaping and refining the guidance at several stages. Although the study did not apply a full co‑design methodology, it used an iterative and stakeholder-informed development process that corresponds with participatory principles described in the literature on guideline development. Camden et al. (50) note that engaging diverse stakeholders through multiple strategies can improve both the quality of research findings and the practical applicability of rehabilitation initiatives. In this study, such multifaceted engagement supported the formulation of guidance that reflects the needs of different stakeholder groups and is therefore pragmatic for use in daily practice.

This study has several limitations. First, because the guidance focuses specifically on stroke and moderate to severe TBI with imaging‑confirmed structural injury, its applicability to other ABI populations may be limited. Second, although a wide range of stakeholders contributed across the different study phases, the Delphi panel and expert participants were recruited from professional networks, which may limit their representativeness. Finally, as the study focused on the development of the guidance, its implementation and effectiveness in clinical practice were not evaluated.

Conversely, it is necessary to discuss a critical commentary on the AQUA principles that were followed while developing the guidance. The AQUA principles value the involvement of various stakeholders in guideline development, but they do not sufficiently account for the differences that exist between representatives of the same target group, nor do they adequately account for attrition over time. The argument put forth is that, for this reason alone (among others), it is beneficial for a guidance to be updated on a regular basis.

The development of an evidence- and consensus-based national guidance aimed at improving the community rehabilitation services for people with ABI proceeded largely according to plan. At the process level, we saw constructive cooperation among the various parties involved. Inclusion of participants for all groups and study phases went seamlessly. The iterative process of providing feedback and adjusting the guidance contributed to a final version that most parties could approve. It is unfortunate that the guidance has not been authorised by the professional association of physiotherapists. However, this does not detract from the content of the guidance, especially considering the process that was followed for its development.

### Recommendations

The process has demonstrated that, in addition to the written consent of stakeholders, there should be very clear agreements on the representation and participation of the different stakeholders beforehand. These agreements should be made prior to applying the AQUA principles [10].

Based on these study findings, and given the practical and societal relevance of the guidance, we recommend the implementation of this guidance within regional primary care networks in the Netherlands. These networks offer existing interdisciplinary infrastructures and collaborative frameworks, making them a suitable starting point for implementation. This recommendation has led to two follow-up projects, for which new grants were obtained: a regional implementation and evaluation study in three Dutch regions, and a national initiative to support dissemination of the guidance through a learning platform that engages local networks and facilitates knowledge exchange.

It is recommended to further elaborate the preliminary framework presented in the guidance, which outlines both overarching competencies and discipline-specific competences for occupational therapists, physiotherapists, and speech and language therapists. This further development of the competency profiles has already been agreed upon and will be led by the professional associations involved. There is also an opportunity to widen the scope of the guidance, as several other health professionals expressed interest in joining the project during the development of the guidance, for example, social workers and psychologists.

The issue of financial compensation for interdisciplinary consultation represents a challenging topic, particularly in terms of its implementation. However, it was brought to the attention of the trade association representing the interests of health insurers during the development of the guidance. This study indicates that interdisciplinary consultation is a crucial aspect of organised collaboration in primary rehabilitation for people with ABI. In the current context, there is a notable absence of financial compensation for allied health professionals, which presents an opportunity for improvement.

## Conclusions

A Dutch national guidance with aimed at improving primary care rehabilitation for people with ABI was developed successfully through an iterative process. The national guidance has been authorised by the professional associations and contains practical tips and advice for implementation by occupational therapists, physiotherapists, and speech and language therapists regarding competencies, the organisation of the right care at the right time within regional primary care networks, and the visibility and accessibility of therapists. Based on this study, we recommend distinguishing between people with different forms of ABI and highlight the need for psychological counselling. The guidance should be considered as a starting point to strengthen primary care rehabilitation for people with ABI. Recently, several initiatives have been launched to supports its implementation.

## Supplementary Information

Supplementary Material 1.

## Data Availability

The datasets used and/or analysed during the current study are available from the corresponding author upon reasonable request.

## Abbreviations

ABI: acquired brain injury
AQUA: Advisory and Expert Group on Quality Standards
DNHI: Dutch National Health Institute
EN: Dutch association of occupational therapy
GP: general practitioner
GRADE: Grading of Recommendations Assessment, Development and Evaluation
KNCN: Dutch Stroke Knowledge Network
KNGF: Dutch Association of Physiotherapy
NVLF: Dutch Association of Speech Therapy
RQ: research question
SANRA: Scale for the Assessment of Narrative Review Articles
TBI: traumatic brain injury
VRA: Dutch Society of Rehabilitation Medicine
WGBO: Medical Treatment Contracts Act
WMO: Dutch Medical Research Involving Human Subjects Act
ZonMw: The Netherlands Organisation for Health Research and Development
