# Supplementary material for: Development of a guidance to improve allied primary healthcare after acquired brain injury in the Netherlands – a mixed-methods study
Source: BMC Prim Care. 2026 Mar 28;27:179. doi: 10.1186/s12875-026-03281-x (PMC13151085; doi:10.1186/s12875-026-03281-x)
Supplement: Supplementary file 1 — Supplementary Material 1. [file 12875_2026_3281_MOESM1_ESM.docx]

**APPENDIX I**

**Topic list for the interviews with experts by experience**

| **Would you like to introduce yourself briefly first?**   - Personal information; name and region - Can you indicate in which way you are an experience expert? - Do you deploy your knowledge as an expert by experience? |  |
| --- | --- |
| **First of all, I am curious if you recognise the problem areas described earlier?**  **Can you tell a bit about your experiences with therapists (occupational therapists, physiotherapists, and speech and language therapists)?**  **When it comes to therapists' knowledge and skills, what could you tell about them?** |  |
| - Do you recognise that therapists are unclear about the necessary knowledge and skills to treat people with brain injury? - What is this lack of clarity about and to whom does it apply? - In your opinion, what makes a therapist competent and competent to treat people with brain injury? Why? - Where are your needs in therapists' knowledge and skills? - To what extent do current professional options match the needs and wishes of people with brain injury? If not, why not? - Have you thought of or found your own solutions to better identify the necessary knowledge and skills of therapists? What are these solutions?   - What do you think are conducive factors in implementing these solutions/directions? Why?   - What do you think are hindering factors in implementing these solutions/directions? Why? | - What exactly do you mean by...? - Can you tell me more about...? - Can you give me an example of...? - I'm not sure I understood you correctly, can you tell me more about that? - I don't know what you mean by .... Can you give me examples? - You told.... Can you tell me more about that? - You just told me about.... I would also like to know about.... - How do you experience it? - What do you think about it? - What does it mean to you? |
| **What can you tell about the coordination and collaboration between different therapists in your area?**   - Could you describe what the right care at the right time means to you? - Do you recognise the mentioned problem of being able to find the right care at the right time? - Can you give examples of regional cooperation and how you experienced this? - Have you found solutions yourself, or have you noticed solutions to be able to offer the right care at the right time within regional cooperation? Which ones?   - What do you think are promoting factors of these solutions/directions? Why?   - What do you think are hindering factors of these solutions/directions? Why? | Examples right care, right time:   - Is it clear to you which caregiver can/should be called in at which time? - Is there communication between the different care providers, and in what way? - Is data and information transferred, and in what way? Does this fit in with the way you want to receive care? - Is there a point of contact/case manager/dashboard within the region? - Who is in charge? Is there joint direction? |
| **The third problem that was outlined is finding the right therapist, both for patients and health professionals. Can you talk a bit about this as well?**   - Have you yourself experienced problems finding the right therapists? If yes, when? - Do you yourself have knowledge of the capabilities of different professionals? - At what point did the problem of finding the right therapists arise? - Did you yourself come up with or find solutions to finding the right therapist? Which ones? - What would the ideal situation for you look like for finding the right therapist?   - What do you think are conducive factors in implementing these solutions/situations? Why?   - What do you think are hindering factors in implementing these solutions/situations? Why? | - Do financial aspects play a role in this? - Does travel distance/accessibility play a role in this? |
| **In your opinion, are there any promoting and hindering factors in the development and implementation of the guidance?** |  |
| **Is there anything else you want to say, or do you want to come back to something?** |  |

**APPENDIX II**

**List of the 74 items presented in the Delphi study.**

| **Therapists' knowledge and skills** |
| --- |
| 1. Specialization in brain injury 2. Generalists 3. Brain injury-specific training 4. Multi-/ interdisciplinary collaboration 5. Transparency in expertise 6. Providing customized care 7. Client-driven approach 8. Cognitive rehabilitation 9. Assistive devices and provisions 10. Coping styles 11. Arm-hand function training 12. Prevention of shoulder complaints 13. Edema prevention (arm/hand) 14. Strength and conditioning 15. Lifestyle advice/ secondary prevention 16. Gait analysis 17. Screening for fall risk 18. Balance training 19. Spasticity treatment 20. Disorder-specific (cognitive-linguistic) aphasia therapy aimed at improving language 21. (Functional) aphasia therapy aimed at improving communication 22. Dysarthria treatment for speech disorders 23. Dysphagia treatment for swallowing disorders 24. Home treatment 25. Treatment in an exercise room or gym 26. Work experience 27. Gaining experience or practice through hands-on work or practical experience 28. Minimal caseload 29. Affinity with brain injury 30. Communication 31. Being able to listen 32. Knowledge networks focused on acquiring and exchanging knowledge 33. Keeping knowledge up to date 34. Referring back to the second/third line |
| **Right care at the right time within regional collaboration** |
| 1. Formal networks at the policy level 2. Experiential networks focused on exchanging experiences 3. Knowledge sharing 4. Informal networks 5. 'Bottom-up' approach in network organization 6. Uniformity in networks 7. Central support for networks 8. Quality certification of networks 9. Network coordinator 10. Communication via the Siilo app (medical messaging service for professionals). 11. Warm hand-over 12. Multidisciplinary consultation 13. Multidisciplinary collaboration 14. Making agreements about collaboration 15. Video calling with the client and healthcare provider for transfer of information 16. Funding for multi/interdisciplinary consultations 17. Brain injury case manager 18. ‘Hub’, coordinator of care |
| **Visibility and accessibility of therapists** |
| 1. Referring from the second/third line to the first line 2. Referring within informal networks 3. Need for a website with therapists 4. Need for a knowledge base 5. Regional decision guide 6. Brain Injury Guide 7. Shortage of specialized occupational therapists 8. Shortage of specialized physiotherapists 9. Shortage of specialized speech therapists 10. Shortage of aphasia therapists |
| **Additional topics** |
| 1. Need for a guidance 2. Implementing the guidance requires a time investment 3. Implementing the guidance requires a financial investment 4. Offering the guidance in a 'blended' format 5. Visual support in the guidance 6. Experiential expertise 7. Willingness to engage in therapy 8. Financial aspects for the client 9. Insurance status of the client 10. Distinguishing between stroke and traumatic brain injury 11. Accessibility by the general practitioner 12. Knowledge level of the general practitioner |

**APPENDIX III**

**Summary – recommendations and practical tips for all key questions**

The present appendix comprises an English-language summary of the recommendations and practical tips for each key question addressed in the original Dutch guidance. It is intended to give international readers a clear impression of the final outcomes of the development process, as well as the overall content and scope of the full guidance.

The guidance was developed as a preliminary framework to support and improve primary allied healthcare for individuals with acquired brain injury. The intended audience for this text encompasses allied health professionals, namely physiotherapists, occupational therapists, and speech and language therapists, in addition to other professionals involved in primary rehabilitation care.

For those seeking more detail, the original Dutch version, which is 24 pages in length, is available via <https://kennisnetwerkcva.nl/wp-content/uploads/2023/05/KNCN-Handreiking-230502-online.pdf>.
